# Validity of Measurement for Trailing Limb Angle and Propulsion Force during Gait Using a Magnetic Inertial Measurement Unit

**DOI:** 10.1155/2019/8123467

**Published:** 2019-12-19

**Authors:** Takasuke Miyazaki, Masayuki Kawada, Yuki Nakai, Ryoji Kiyama, Kazunori Yone

**Affiliations:** ^1^Graduate School of Health Sciences, Kagoshima University, 8-35-1 Sakuragaoka, Kagoshima City, Kagoshima 890-8544, Japan; ^2^Department of Rehabilitation, Tarumizu Municipal Medical Center, Tarumizu Central Hospital, 1-140 Kinkocho, Tarumizu City, Kagoshima 891-2124, Japan; ^3^Course of Physical Therapy, School of Health Sciences, Faculty of Medicine, Kagoshima University, 8-35-1 Sakuragaoka, Kagoshima City, Kagoshima 890-8544, Japan

## Abstract

Propulsion force and trailing limb angle (TLA) are meaningful indicators for evaluating quality of gait. This study examined the validity of measurement for TLA and propulsion force during various gait conditions using magnetic inertial measurement units (IMU), based on measurements using a three-dimensional motion analysis system and a force platform. Eighteen healthy males (mean age 25.2  ±  3.2 years, body height 1.70   ±  0.06 m) walked with and without trunk fluctuation at preferred, slow, and fast velocities. IMU were fixed on the thorax, lumbar spine, and right thigh and shank. IMU calculated the acceleration and tilt angles in a global coordinate system. TLA, consisting of a line connecting the hip joint with the ankle joint, and the laboratory's vertical axis at late stance in the sagittal plane, was calculated from thigh and shank segment angles obtained by IMU, and coordinate data from the motion analysis system. Propulsion force was estimated by the increment of velocity calculated from anterior acceleration measured by IMU fixed on the thorax and lumbar spine, and normalized impulse of the anterior component of ground reaction force (AGRF) during late stance. Similarity of TLA measured by IMU and the motion analysis system was tested by the coefficient of multiple correlation (CMC), intraclass correlation coefficient (ICC), and root mean square (RMS) of measurement error. Relationships between normalized impulse of AGRF and increments of velocity, as measured by IMU, were tested using correlation analysis. CMC of TLA was 0.956–0.959. ICC between peak TLAs was 0.831–0.876 (*p* < 0.001), and RMS of error was 1.42°–1.92°. Velocity increment calculated from acceleration on the lumbar region showed strong correlations with normalized impulse of AGRF (*r* = 0.755–0.892, *p* < 0.001). These results indicated a high validity of estimation of TLA and propulsion force by IMU during various gait conditions; these methods would be useful for best clinical practice.

## 1. Introduction

Gait ability is a fundamental function in activities of daily living and associates with risk of fall and the capacity to be mobile within the community [1–4]. It is important to evaluate gait ability during rehabilitation in relation to dysfunction in the lower limbs [5, 6]. In rehabilitation, therapists guide patients to achieve a more efficient walking pattern; consequently, an objective and simple method to assess the gait quality and characteristics is necessary for gait training.

Ground reaction force (GRF) is often used to assess quality of gait. The anterior component of GRF (AGRF) during late stance is an important element of propulsion force. Impulse of AGRF in late stance is usually used to estimate the propulsion force and increases velocity of center of mass during late stance [7–9]. Previous studies report that propulsion force correlates with hip extension angle [10–12] as well as hip flexion and ankle plantar flexion moments [13]. In addition, trailing limb angle (TLA), an angle consisting of a line connecting the hip joint with the ankle joint, and the laboratory's vertical axis at late stance in the sagittal plane [14], is shown to be related to AGRF [15, 16]. TLA is the main contributor to an increase in propulsion force following training [17] and is changed by gait training [18–21]. In addition, TLA correlates with lower limb kinetics and ability to walk long distance [15, 22, 23]. Therefore, propulsion force and TLA would be meaningful indicators, representing the kinetics and kinematics of gait quality during gait rehabilitation.

TLA and propulsion force have been measured using a three-dimensional motion analysis system and a force platform in previous studies [14, 16, 24]. However, neither have ever been utilized in clinical practice because of complications in measurement and data processing. Conversely, wearable sensors including magnetic and inertial measurement units (IMU), permitting the objective and simple assessment of human movement, have become widely used tools in motion analysis [25–30]. TLA might be estimated from thigh and shank segment angles obtained by IMU. Meanwhile, the impulse (*I*) is equal to the average force (*F*_*average*_) multiplied by the duration the force act (Δ*t*) and to the body mass (*m*) multiplied by the change in velocity during the force act (Δ*v*), as the following equation: *I* = *F*_*average*_Δ*t* = *ma*_*average*_Δ*t* = *m*Δ*v*, where *a*_*average*_ is the average acceleration during the force act. Impulse divided by body mass is equal to the change in velocity. Therefore, increment of velocity of trunk during late stance calculated from anterior acceleration obtained by IMU should also correlate with impulse of AGRF normalized by body mass and could be used as an indicator for propulsion force. However, few studies have reported TLA and velocity increment of trunk during late stance by IMU.

The aim of this study was to determine the validity of measurement for TLA and propulsion force during various gait conditions using IMU, using the three-dimensional motion analysis system and force platform as gold standards. We hypothesized that the TLA, as measured by IMU, and the three-dimensional motion analysis system are similar, and that an increment of velocity of the trunk calculated from acceleration measured by IMU would correlate with the normalized impulse of the AGRF. During several velocities, we examined both upright gait and gait with forward trunk lean during early stance because gait velocity and trunk angle in the sagittal plane affect TLA and AGRF. These findings will be able to provide basic information on gait analysis using IMU in clinical practice.

## 2. Materials and Methods

### 2.1. Subjects

Eighteen healthy males (age, 25.2 ± 3.2 y; height, 1.70 ± 0.06 m; body mass, 62.7 ± 6.1 kg; average ± standard deviation) without orthopedic or neurological disorders participated in this study. Prior to the investigation, all participants provided written informed consent for participation in the study. This study was approved by the Ethics Committee on Epidemiological Studies of Kagoshima University (number, 170167Epi).

### 2.2. Measurement

In this study, we tested the validity of TLA and propulsion force measurement using IMU based on calculations obtained by three-dimensional motion analysis system and a force platform. TLA and propulsion force of the right lower extremity were analyzed in this study.

**Figure 1:**
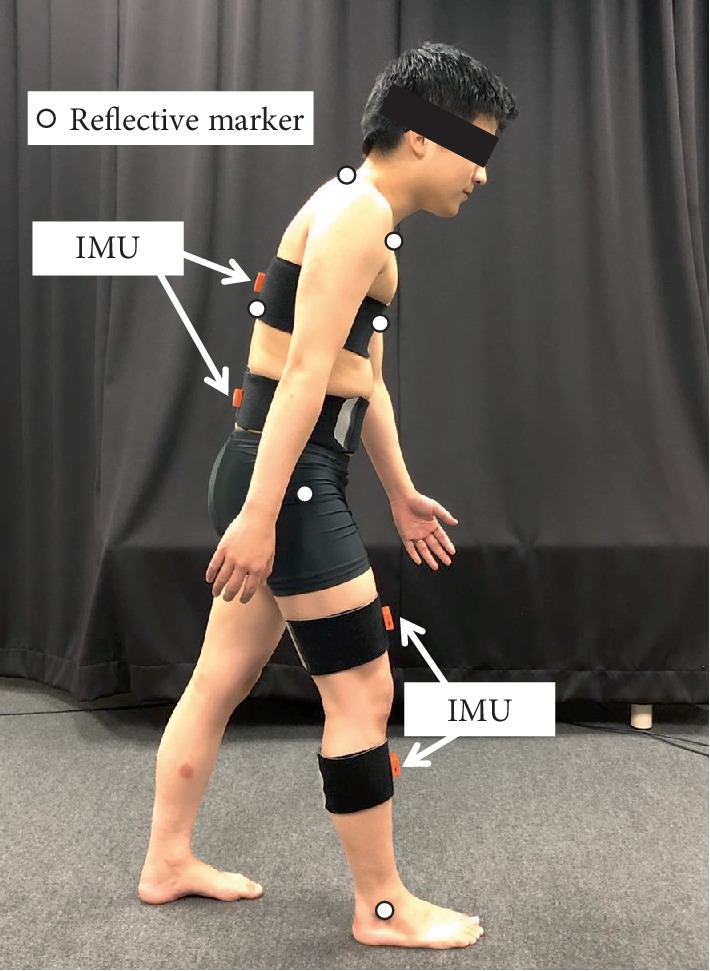
Position of reflective markers and magnetic inertial measurement units (IMU); subject shown in gait with forward lean during right early stance.

Four IMU (MTw Awinda, Xsens, Enschede, NL), a three-dimensional motion analysis system consisting of six infrared cameras (VICON, Oxford Metrics, Oxford, UK) and a force plate (BP600400, AMTI, MA, USA) were used to obtain kinematics and kinetics data during gait. These were synchronized with each other. Our sampling frequency was 100 Hz on IMU and cameras and 1000 Hz on the force plate. IMU consisted of a 3D rate gyroscope, 3D accelerometer, and 3D magnetometer. MT manager (4.7.2, Xsens, Enschede, NL) was used to obtain 3-axis acceleration and tilt angles in a global coordinate system, that software used the Kalman filter for estimating those from magnetic and inertial data. Reliability of IMU and the software have been previously reported [31]. Prior to gait analysis, reflective markers were attached with adhesive tape to the jugular notch, xiphoid process, spinous processes of C7 and T10, the right greater trochanter of the femur, and the lateral malleolus of the fibula (Figure 1). IMU were fixed by elastic belts on the posterior thorax (T7), lumbar spine (L3), and right anterior thigh and shank. IMU were attached along the frontal plane where possible and calibrated so that the vertical direction of the IMU coordinate system lay in the same direction as gravity during relaxed standing by MT manager.

Participants walked 10 m under 5 conditions: without forward trunk lean at preferred velocity (Preferred), slow velocity (Slow), fast velocity (Fast), gait with forward trunk lean during right early stance at preferred velocity (Lean-preferred), and slow velocity (Lean-slow). Forward trunk lean during early stance was adopted because of its prevalence in stroke patients and effect on AGRF and TLA [32]. Instructions were provided verbally: “walk as usual” in Preferred; “walk faster (or Slower) than usual” in Fast and Slow [33]; “walk with a forward trunk lean when your right heel contacts the floor and then return to upright, in usual velocity (Lean-preferred) or in slow velocity (Lean-slow)” (Figure 1). The starting position was standardized to ensure that each participant would step onto the force plate with the right foot first. The measurement was performed 5 times under each walking condition following an appropriate warm-up period for practice, and several minutes of rest was allowed between each walking condition. Each walking condition was measured in ascending order of random numbers generated by Microsoft Excel RAND function.

### 2.3. Data Analysis

The central one stance phase from each trial was analyzed. A third-order Butterworth low-pass filter was performed on kinematic data measured by the motion analysis system and IMU with a 10 Hz cut-off frequency, and on GRF and acceleration by IMU with a 20 Hz cutoff frequency to reduce the noise.

**Figure 2:**
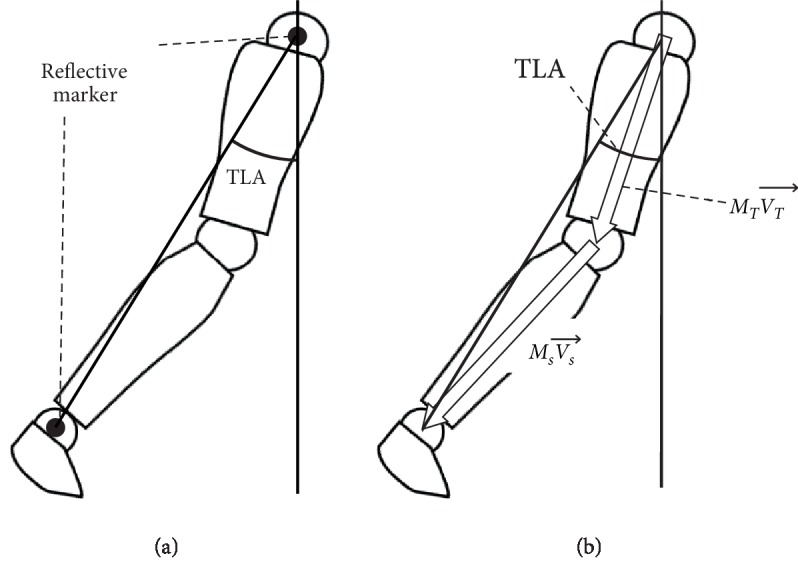
Estimation of the trailing limb angle (TLA) by motion analysis system (a) and magnetic inertial measurement units (b). *M*_*T*_, tilt angle matrix of the thigh segment; *M*_*s*_, tilt angle matrix of the shank segment; $\overset{\to }{V_{T}}$, vector of the thigh segment; $\overset{\to }{V_{s}}$, vector of the shank segment.

TLA was calculated as an angle consisting of the laboratory's vertical axis and a line connecting the lateral malleolus with the greater trochanter in the sagittal plane from the coordinate data measured by the motion analysis system, according to a previous study [14] (Figure 2(a)). Prior to estimation of TLA using IMU, location of the knee joint and the ankle joint relative to the hip joint was estimated from the tilt angle matrix measured by IMU and the vector of the thigh and shank segment coordinated by the segment length (Figure 2(b)). To minimize the error caused by the mounting angle of the sensor, data from the IMU attached to the thigh and shank were calibrated in the horizontal plane [34] with the following equation:

(1) $$\begin{array}{c} M_{Calibrated}=M_{IMU}∙{\left(M_{Diff}\right)}^{T}, \end{array}$$

(2) $$\begin{array}{c} M_{Seg}={\left(M_{Offset}\right)}^{T}∙M_{Calibrated}, \end{array}$$

where *M*_*IMU*_ is the rotation matrix obtained by IMU and (*M*_*Diff*_)^*T*^ is estimated so that the trajectory of the knee and ankle joint, as measured by IMU during gait, corresponded to forward direction in the horizontal plane using principal component analysis, and (*M*_*Offset*_)^*T*^ is obtained to adjust *M*_*Calibrated*_ to measure zero at quiet standing, and *M*_*Seg*_ is the rotation matrix of thigh and shank segment [34]. Then, TLA was calculated from the location of the ankle joint relative to the hip joint in the sagittal plane, in a similar manner to the TLA measured by the motion analysis system (Figure 2(b)). The TLA measured by IMU and the motion analysis system were adjusted to measure zero at quiet standing. Maximum TLA at pre-swing was calculated as the representative value.

**Figure 3:**
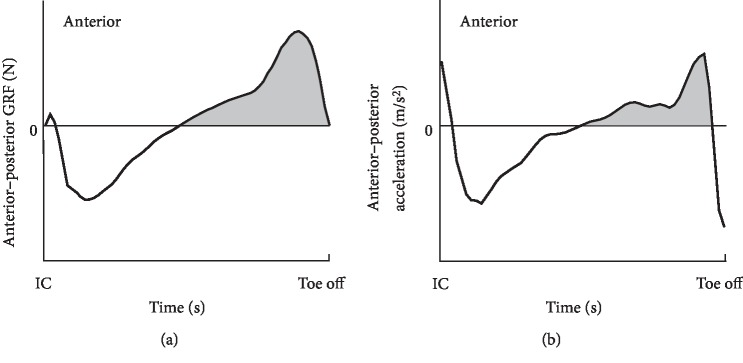
Waveform of the anterior–posterior component of ground reaction force (GRF) (a) and the anterior–posterior acceleration of lumbar spine (b) during stance phase. Late stance was defined as duration that the anterior–posterior GRF shows anterior force. The intervals of anteriorly directed GRF and acceleration (the gray area) were integrated to estimate the impulse of the anterior GRF and the velocity increment of trunk (thorax, lumbar spine). IC, initial contact.

The time to peak TLA was defined as the duration from right initial contact to maximum TLA at pre-swing. The trunk lean angle was calculated as the Euler angle of the plane measured by markers placed on the jugular notch, xiphoid process, and the spinous processes of C7 and T10 in a global coordinate system by Nexus 1.8.5 (VICON, Oxford Metrics, Oxford, UK). In addition, the time integral of AGRF measured from the force plate and anterior acceleration measured by the IMU fixed to the thorax and lumber supine during late stance, defined as duration the anterior–posterior components of GRF showed anterior force, were calculated as an indicator of propulsion force (Figure 3). Those represent the impulse of AGRF and increment of velocity of trunk, respectively. Impulse of AGRF was normalized by body mass. In addition, maximum anterior acceleration of the center of mass, generated by AGRF, was calculated by dividing AGRF by the body mass. Then, the maximum anterior acceleration estimated by IMU and AGRF was compared. Data processing was performed using MATLAB R2017b (MathWorks Inc, MA, USA) mathematical software.

### 2.4. Statistical Analysis

The average of maximum value and time to peak of TLA, and the maximum anterior acceleration measured by both means, increment of velocity by IMU, normalized impulse of AGRF, gait velocity, and maximum trunk-lean angle at early stance were calculated from 5 repetition trials as the representative value. Differences dependent on gait conditions were analyzed by one-way repeated-measures analysis of variance (ANOVA). The similarity of waveform of both TLAs was evaluated using coefficient of multiple correlation (CMC) from data at 250 ms before and after toe-off according to a previous study [35, 36]. CMC was classified as moderate (0.65–0.75), good (0.75–0.85), very good (0.85–0.95), or excellent (0.95–1). The coincidence of peak value and time to peak of TLA measured using IMU and motion analysis system was tested by intraclass correlation coefficient type 2.1 (ICC_(2,1)_), and root mean square (RMS) of measurement error. ICC was classified as slight (0.0–0.20), fair (0.21–0.40), moderate (0.41–0.60), substantial (0.61–0.80), or almost perfect (0.81–1.00) [37].

Additionally, to test the validity of measurement for propulsion force using IMU, relationships between the increment of velocity by IMU and the normalized impulse of AGRF, and the maximum anterior acceleration by IMU and AGRF were analyzed using Pearson's correlation coefficient. Statistical analyses were performed using SPSS 25 (IBM, NY, USA), and the threshold of significance was set at 5%.

## 3. Results

### 3.1. Gait Characteristics

Gait speed was 1.23 ± 0.17 m/s at Preferred, 0.97 ± 0.16 m/s at Slow, 1.53 ± 0.26 m/s at Fast, 0.97 ± 0.13 m/s at Lean-preferred, and 0.76 ± 0.15 m/s at Lean-slow; gait speed differed significantly between gait conditions (F_(2.4, 40.5)_ = 95.5, *p* < 0.001). Meanwhile, peak trunk anterior lean angles during stance phase at Lean-preferred (26.6 ± 14.4°) and Lean-slow (23.1 ± 7.2°) were larger than those of Preferred gait (5.7 ± 4.8°) and Slow gait (6.3 ± 7.1°; *F*_(2.3, 39.0)_ = 50.0, *p* < 0.001). These results indicated that participants could simulate abnormal gait according to our instruction.

### 3.2. Similarity of TLA Measured by IMU and Motion Analysis System

**Figure 4:**
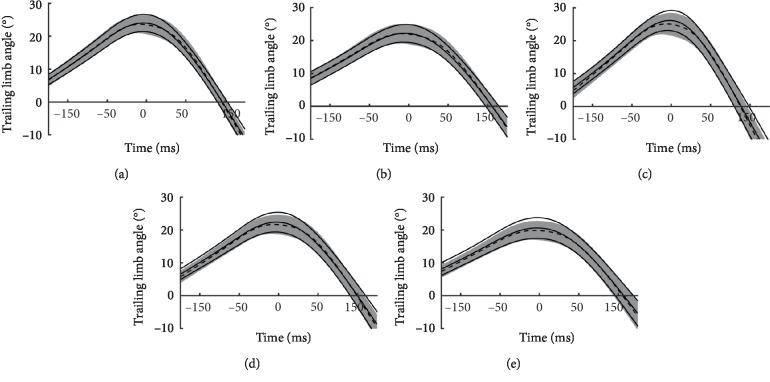
Trailing limb angle during 250 ms before and after toe-off measured by magnetic inertial measurement units (IMU) and a motion analysis system. Coefficient of multiple correlation was 0.959 in Preferred (a), 0.959 in Slow (b), 0.958 in Fast (c), 0.957 in Lean-preferred (d), and 0.956 in Lean-slow (e), respectively. The broken line and the gray area indicate the mean and one standard deviation for IMU. The solid line indicates the mean and one standard deviation for motion analysis system. The zero of time indicates the right toe-off. Each average and standard deviation represented for 18 participants.

The time series of TLA measured by IMU and the motion analysis system from late stance to initial swing were very similar to each other in all gait conditions, and CMC ranged from 0.956 to 0.959, representing very good or excellent similarity (Figure 4). Both TLA showed peak at around toe-off, and peak differences were very small; angle peaks were 20.6–26.3° and 20.0–25.5° in IMU and motion analysis system, respectively. ICC_(2,1)_ between both TLAs was 0.831–0.876, and RMS of error was 1.42–1.92°, indicating moderate to high agreement in all gait conditions (*p* < 0.001, Table 1). Similarly, ICC_(2,1)_ of duration from initial contact to peak of TLA was 0.876–0.992, and RMS of error was 14.8–16.7 ms, indicating high agreement in all gait conditions (*p* < 0.001, Table 2).

### 3.3. Relationship between Increment of Velocity Calculated from Acceleration Measured by IMU, Normalized Impulse of AGRF, and Maximum Acceleration by IMU and AGRF

Increment of velocity calculated from acceleration measured by IMU on the thorax and lumbar spine correlated strongly with normalized impulse of AGRF measured by the motion analysis system except for thorax acceleration in the Lean-preferred condition (*r* = 0.755–0.892, *p* < 0.001; Table 3). Increment of velocity of the thorax in the Lean-preferred condition and normalized impulse of AGRF showed moderate correlation (*r* = 0.581, *p* = 0.012). Maximum anterior acceleration obtained by IMU and AGRF showed more moderate relationship except for thorax in the Slow condition (*r* = 0.484–0.828, *p* < 0.042; Table 4).

## 4. Discussion

We examined the validity of TLA and velocity increment of trunk by IMU during various walking conditions, based on a motion analysis system. The present study showed that IMU could validly estimate the TLA; consistent with our hypothesis, a significant relationship was found between increments of velocity calculated from acceleration measured by IMU and normalized impulse of AGRF. Also, a significant relationship was found between acceleration obtained by IMU and AGRF. Our findings suggested that IMU would be useful during clinical practice in estimating the TLA and propulsion force during gait.

A time series of TLA measured by IMU and motion analysis system showed very good agreement, and CMC ranged from 0.956 to 0.959, consistent with a previous study [25]. In addition, good agreement was observed in the peak value and time to peak of TLA measured by both means; ICC was greater than 0.831 in peak value and was greater than 0.876 in time to peak. RMS of TLA error was also small, ranging from 1.42° to 1.92°. These results indicated the validity of gait measurement using IMU. Although we found no literature analyzing TLA using IMU, several studies compared the joint angle during gait measured by IMU and optical motion analysis system; these studies reported that RMS of error of the flexion–extension angle was 3.1–8.7 for the hip joint and was 2.7–6.8° for the knee joint [34, 38, 39]. Our results were equal to or better than the results of these previous studies.

Sensor accuracy has been rapidly improving in recent years, and the dynamic absolute accuracy in the absolute angle of IMU used in this study was 0.5° at 90°/s and 1.8° at 180°/s, according to a previous study tested using the bench test [31]. The high accuracy of IMU contributed to our results. In addition, calibrating to minimize the error caused by the sensor mounting contributed to measurement accuracy using IMU [34]. Meanwhile, the accuracy of IMU was affected by motion velocity [31]: an increase in motion velocity reduced the accuracy of IMU and increased the soft-tissue artifact caused by its weight. Nevertheless, good agreement was found between TLA measured by IMU and the motion analysis system in fast gait, with an average velocity of 1.53 m/s. This result indicates that the effect of gait velocity on decrease in accuracy of gait measurement using IMU would be small in clinical practice for those patients with a neurological disorder or motor dysfunction who walk slowly.

A significant relationship was found between the increments of velocity calculated from acceleration of trunk and normalized impulse of AGRF during gait. In addition, similar relationship was observed between acceleration of thorax and lumbar segment obtained by IMU, and acceleration of the center of mass calculated by AGRF. Although previous studies reported that vertical acceleration correlated with vertical trunk acceleration during gait [40, 41], no literature investigated the estimation of AGRF by IMU. By Newtonian equation of motion, GRF expresses gravity and acceleration acting on a center of mass, and the impulse divided by body mass indicates the change in velocity. Thus, with the trunk being the heaviest segment of the human body, the increment of velocity calculated from acceleration of the thorax and lumbar could evaluate the acceleration of center of mass and AGRF [42]. We treated the acceleration in a global frame, which consisted of adjusted raw data from the local sensor frame, according to the absolute angle. High accuracy of the absolute angle of IMU would contribute to the precise adjustment of acceleration in the global frame. However, breaking GRF acting on contralateral leg would weaken that relationship. And, that relationship was lower during the gait condition of forward trunk lean in thorax acceleration. Overestimation or underestimation of upper trunk acceleration caused by anteroposterior trunk fluctuation reduced measurement accuracy of propulsion force as recorded by the thorax sensor. Therefore, increment of velocity calculated from acceleration of the lumbar spine was useful for evaluation of propulsion force during gait.

One limitation of our study was that we tested the validity of TLA and propulsion force measured by IMU for able-bodied male subjects only. Movements and stability of trunk during gait are affected by aging, gender, and pathologies [43, 44]. Therefore, further study could focus on a broader range of male and female subjects, including older people, and patients with neurological disorders or motor dysfunction in order to better utilize gait analysis by IMU in clinical practice. On a further note, several previous studies investigate the collection of key kinematics and kinematic information, including joint angle, trajectory of center of mass, ground reaction force, during gait, and so on using the smallest possible inertial sensors [41, 45]. Meanwhile, gait training using biofeedback of propulsion force on gait velocity and TLA could improve gait performance [18, 46–48]; however, these methods are not easily reproduced in clinical practice due to their complicated environmental restrictions. The findings of this study can contribute towards establishing a more clinically friendly method for gait assessment as well as biofeedback gait training in clinical practice.

## 5. Conclusions

This study showed the excellent validity of the evaluation of TLA and propulsion force by IMU under several gait conditions; their portability and affordability make them highly effective tools for clinical gait assessment. Our results propose the use of IMU for gait analysis in clinical practice.

## Figures and Tables

**Table 1:** Peak of trailing limb angle measured by magnetic inertial measurement units (IMU) and motion analysis system (mean ± SD).

	IMU (°)	Motion analysis system (°)	ICC (95% CI)	*p*	RMS (°)
Preferred	24.1 ± 2.6	23.9 ± 2.6	0.836 (0.617–0.935)	<0.001	1.50
Slow	22.3 ± 2.7	22.1 ± 3.0	0.876 (0.699–0.952)	<0.001	1.42
Fast	26.3 ± 3.3	25.5 ± 3.3	0.833 (0.597–0.935)	<0.001	1.92
Lean-preferred	22.5 ± 3.0	21.8 ± 2.9	0.831 (0.599–0.933)	<0.001	1.74
Lean-slow	20.6 ± 3.2	20.0 ± 2.8	0.851 (0.645–0.942)	<0.001	1.64

**Table 2:** Time to peak of trailing limb angle measured by magnetic inertial measurement units (IMU) and motion analysis system (mean ± SD).

	IMU (ms)	Motion analysis system (ms)	ICC (95% CI)	*p*	RMS (ms)
Preferred	629.2 ± 29.9	620.9 ± 30.0	0.876 (0.631–0.956)	<0.001	15.5
Slow	737.9 ± 66.3	733.9 ± 70.1	0.978 (0.943–0.992)	<0.001	14.8
Fast	566.0 ± 49.4	557.3 ± 46.6	0.944 (0.816–0.981)	<0.001	16.7
Lean-preferred	754.0 ± 60.7	750.6 ± 63.7	0.971 (0.926–0.989)	<0.001	15.4
Lean-slow	904.4 ± 115.1	898.8 ± 116.4	0.992 (0.978–0.997)	<0.001	15.0

**Table 3:** Relationship between increments of velocity calculated from acceleration of trunk (thorax, lumbar spine) and normalized impulse of anterior ground reaction force (AGRF) (mean ± SD).

	Thorax		Lumbar		Normalized impulse of AGRF (N·s/kg)
	Increments of velocity (m/s)	*r* (*p*)	Increments of velocity (m/s)	*r* (*p*)	
Preferred	0.183 ± 0.052	0.781 (<0.001)	0.233 ± 0.065	0.757 (<0.001)	0.287 ± 0.052
Slow	0.176 ± 0.068	0.790 (<0.001)	0.213 ± 0.057	0.816 (<0.001)	0.264± 0.052
Fast	0.202 ± 0.081	0.848 (<0.001)	0.271 ± 0.099	0.755 (<0.001)	0.298 ± 0.057
Lean-preferred	0.193 ± 0.058	0.581 (0.012)	0.273 ± 0.087	0.809 (<0.001)	0.289 ± 0.067
Lean-slow	0.216 ± 0.095	0.807 (<0.001)	0.285 ± 0.115	0.892 (<0.001)	0.286 ± 0.092

**Table 4:** Relationship between acceleration of trunk (thorax, lumbar spine) obtained by magnetic inertial measurement unit and acceleration of the center of mass calculated from anterior ground reaction force (AGRF) (mean ± SD).

	Thorax		Lumbar		AGRF
	Acceleration (m/s^2^)	*r* (*p*)	Acceleration (m/s^2^)	*r* (*p*)	Acceleration (m/s^2^)
Preferred	1.97 ± 0.56	0.484 (0.042)	3.12 ± 0.67	0.590 (0.010)	2.14 ± 0.38
Slow	1.72 ± 0.66	0.445 (0.064)	2.49 ± 0.65	0.680 (0.002)	1.66 ± 0.37
Fast	2.51 ± 0.89	0.770 (<0.001)	3.57 ± 1.01	0.699 (0.001)	2.53 ± 0.48
Lean-preferred	1.72 ± 0.47	0.525 (0.025)	2.48 ± 0.48	0.541 (0.020)	1.70 ± 0.37
Lean-slow	1.49 ± 0.42	0.684 (0.002)	2.05 ± 0.51	0.828 (<0.001)	1.38 ± 0.44

## Data Availability

The data used to support the findings of the current study are available from the corresponding author upon request.
